# Inactivated Poliovirus Vaccine: Recent Developments and the Tortuous Path to Global Acceptance

**DOI:** 10.3390/pathogens13030224

**Published:** 2024-03-04

**Authors:** Roland W. Sutter, Martin Eisenhawer, Natalia A. Molodecky, Harish Verma, Hiromasa Okayasu

**Affiliations:** 1 Tamayo Federal Solutions LLC, Virginia Beach, VA 23452, USA; 2 Polio Eradication Department, World Health Organization, 1211 Geneva, Switzerland; eisenhawerm@who.int (M.E.); vermah@who.int (H.V.); 3 Polio Surge Capacity Support Program, The Task Force for Global Health, Inc., Decatur, GE 30030, USA; nmolodecky@gmail.com; 4 Division of Healthy Environments and Population, Regional Office for the Western Pacific, World Health Organization, Manila 1000, Philippines

**Keywords:** inactivated poliovirus vaccine (IPV), Global Polio Eradication Initiative (GPEI), mucosal immunity, fractional-dose IPV (fIPV)

## Abstract

Inactivated poliovirus vaccine (IPV), available since 1955, became the first vaccine to be used to protect against poliomyelitis. While the immunogenicity of IPV to prevent paralytic poliomyelitis continues to be irrefutable, its requirement for strong containment (due to large quantities of live virus used in the manufacturing process), perceived lack of ability to induce intestinal mucosal immunity, high cost and increased complexity to administer compared to oral polio vaccine (OPV), have limited its use in the global efforts to eradicate poliomyelitis. In order to harvest the full potential of IPV, a program of work has been carried out by the Global Polio Eradication Initiative (GPEI) over the past two decades that has focused on: (1) increasing the scientific knowledge base of IPV; (2) translating new insights and evidence into programmatic action; (3) expanding the IPV manufacturing infrastructure for global demand; and (4) continuing to pursue an ambitious research program to develop more immunogenic and safer-to-produce vaccines. While the knowledge base of IPV continues to expand, further research and product development are necessary to ensure that the program priorities are met (e.g., non-infectious production through virus-like particles, non-transmissible vaccine inducing humoral and intestinal mucosal immunity and new methods for house-to-house administration through micro-needle patches and jet injectors), the discussions have largely moved from whether to how to use this vaccine most effectively. In this review, we summarize recent developments on expanding the science base of IPV and provide insight into policy development and the expansion of IPV manufacturing and production, and finally we provide an update on the current priorities.

## 1. Introduction

In 1988, the World Health Assembly (WHA), the governing body of the World Health Organization (WHO), resolved to eradicate poliomyelitis by the year 2000, and the Global Polio Eradication Initiative (GPEI) was formed [1]. Since the resolution, substantial progress toward eradication has been achieved. However, the eradication target remains elusive [2].

The first vaccine licensed for use against poliovirus was the inactivated poliovirus vaccine (IPV), developed by Jonas Salk, and licensed in the United States in 1955 [3]. Thereafter, IPV was widely used in routine immunization (RI) of infants and children and in campaigns covering all age groups to control poliomyelitis. In the late 1950s, a resurgence of paralytic cases led to concern regarding IPV effectiveness. Subsequently, oral poliovirus vaccine (OPV) was licensed in the United States starting in 1961 and replaced IPV as the vaccine of choice for poliomyelitis prevention. Only two countries (Iceland and Sweden) never used OPV [4]. Improvements in the potency of IPV by a factor of 10 in the 1960s [5,6] (i.e., enhanced-potency IPV), and progress toward eradication, led to a re-evaluation of IPV use and subsequent re-introduction into RI across North America and Western Europe in the early 2000s. Despite its high efficacy at preventing paralysis, the main limitation of IPV was perceived to be its inability to stop poliovirus from spreading among children.

OPV became the main vaccine of choice for the GPEI due to its ease of use, low cost and ability to induce mucosal immunity, required for preventing transmission [7,8]. Until 2005, when monovalent oral poliovirus vaccine type 1 (mOPV1) and type 3 (mOPV3) were licensed, the GPEI used exclusively trivalent OPV (tOPV), decreasing the number of polio-endemic countries from >125 in 1988 to 6 (Afghanistan, India, Pakistan, Egypt, Nigeria and Niger) [9]. In 2005, Egypt and Niger interrupted endemic transmission, leaving only four polio endemic countries. Despite the tremendous impact of OPV, it was well understood that the use of OPV (comprised of live attenuated Sabin strains) would eventually need to be stopped due to the genetic instability of the Sabin strains. Prolonged replication in an otherwise healthy B-cell deficient vaccinee or circulation in an under-vaccinated population would result in mutation and recombination that ended with the emergence of new polioviruses that were as neurovirulent and transmissible as wild poliovirus (having lost all attenuation mutations) [10]. As long as Sabin strains were introduced into communities, the emerging virus could seed new endemics and epidemic circulation, negating the achievements of the eradication effort, and thus be incompatible with eradication.

In November 2012, the Strategic Advisory Group of Experts (SAGE) on Immunization endorsed the Strategic Plan of Action for Polio Eradication 2013–2018 that called for the withdrawal of Sabin strains from the OPV, starting with Sabin type 2 (OPV2) [11]. OPV2 was selected because indigenous wild poliovirus type 2 (WPV2) was last detected in 1999 in Northern India, and OPV2 continued to cause a predictable burden of vaccine-associated paralytic poliomyelitis (VAPP) [12] that became increasingly unacceptable to parents and health providers. Moreover, outbreaks of type-2 vaccine-derived poliovirus (VDPV2) were reported each year, due to continued OPV2 use in populations with sub-optimal immunity. The GPEI coordinated the global cessation of OPV2 in April 2016 [13].

**Figure 1:**
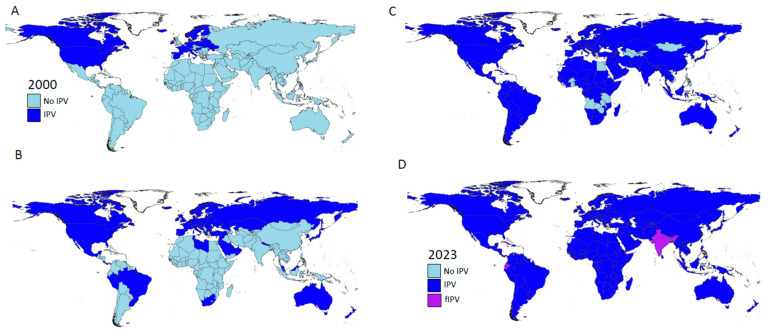
Global map: Countries using IPV/fIPV in routine immunization in: (**A**) 2000; (**B**) 2014; (**C**) 2016; and (**D**) 2023. Source: IPV introduction date: WHO Immunization, (https://immunizationdata.who.int/listing.html?topic=vaccine-intro&location=; accessed on 17 December 2023). fIPV countries [16].

To mitigate the growing susceptibility gap, the GPEI recommended ≥1 dose of IPV to be introduced into RI programs globally in advance of OPV2 withdrawal [11,14]. Figure 1 displays the countries using IPV in 2000, 2016 and 2023. While the majority of countries successfully introduced IPV into RI, due to supply constraints, >43 million children did not receive IPV after OPV2 cessation. In addition, supply constraints limited the use of IPV in outbreak response to cVDPV2 outbreaks, and in 2017, the endorsement for its use in cVDPV2 outbreak response was removed. By 2020, IPV supply constraints were addressed and the GPEI adopted a two-dose IPV schedule for RI [15], in addition to the catch-up IPV vaccination for children that had not received IPV after OPV2 cessation. In 2023, the SAGE restored the recommendation for IPV use in cVDPV2 outbreak response.

To reach a point wherein global technical advisory bodies could recommend IPV first as a risk mitigation tool in 2012 [11] and then as the primary tool for poliomyelitis prevention in 2020 [15] required a massive program of work with IPV, focusing on scientific knowledge, programmatic use, developing safer IPV seed strains and facilitating the expansion of IPV production. This program of work is not yet completed and focuses on non-infectious IPV production, hexavalent vaccine and new administration tools.

In this review, we summarize the recent developments on expanding the science base of IPV and provide insight into policy development and the expansion of IPV manufacturing and production, and finally we provide an update on the current priorities. Thus, we have organized this review into four sections: (1) increasing the scientific knowledge base of IPV; (2) translating new insights and evidence into programmatic action; (3) expanding the IPV supply base for global demand; and (4) continuing to pursue an ambitious research program to develop more immunogenic, easier to administer and safer-to-produce vaccines.

## 2. Increasing the Scientific Knowledge Base of IPV

This chapter focuses on three critical areas of research: (1) better characterization of IPV-induced or -boosted intestinal mucosal immunity; (2) reduction in the number of IPV doses and antigen content to induce immunity response against poliomyelitis; and (3) understanding IPV’s potential in inducing herd immunity.

**Figure 2:**
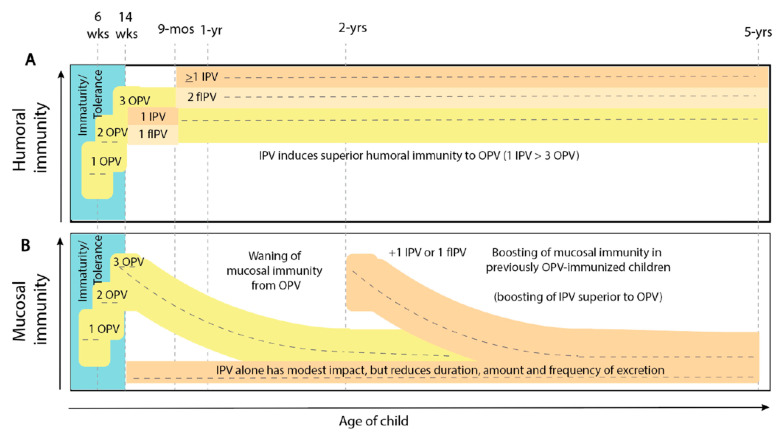
Immunity diagram. (**A**) Humoral immunity. (**B**) Mucosal immunity.

The first area of the IPV program-of-work was to better characterize its contribution to intestinal mucosal immunity against poliovirus (Figure 2).

IPV has been shown to not directly induce intestinal mucosal immunity, even with a mucosal adjuvant. Initial attempts to use a mucosal adjuvant (dmLT-double-mutant heat labile E Coli toxin) to induce mucosal immunity after IPV vaccination were unsuccessful [17,18]. IPV does appear to induce pharyngeal mucosal immunity [19], although the evidence is limited.

While IPV on its own in naïve infants does not induce intestinal mucosal immunity, an ever-increasing body of evidence supports that IPV-vaccinated infants excrete poliovirus for significantly shorter periods and with a lower quantity (lower viral titer) after challenge with vaccine virus or natural infection [20] (Table 1). Efforts to quantify this effect suggested a 95% decrease in excreted virus following challenge in IPV vaccinated persons [7,21]. The shorter excretion period was confirmed in Sweden [22] and the decrease in excreted titer in IPV-vaccinated following a tOPV challenge in Cuba [23]. Moreover, a recent study from Bangladesh further demonstrated that the addition of a dose of IPV can decrease the excretion of OPV in a routine schedule [24]. It has been suggested that IPV potentially produces a priming effect leading to faster response. Therefore, while IPV on its own in naïve infants does not stop transmission, it may reduce transmission by inducing a degree of intestinal mucosal immunity, thereby decreasing the amount and duration of viral shedding.

In contrast to the modest direct impact on intestinal mucosal immunity in naïve infants, IPV has a substantial impact on boosting intestinal mucosal immunity in previously OPV immunized children. Two landmark studies in India demonstrated that IPV could boost intestinal mucosal immunity in OPV-vaccinated children, across age groups from 6-month to 10-year-olds [30,31]. The boosting by IPV on waned mucosal immunity in previously OPV immunized individuals after a bOPV (types 1 + 3) challenge dose was shown to be greater than an additional dose of bOPV. Moreover, a single fractional IPV (fIPV) dose is as effective as a full dose of IPV for boosting previously OPV immunized individuals. This indicates that IPV (and fIPV) can help interrupt transmission in settings with previous OPV use by boosting intestinal mucosal immunity, better than an additional dose of OPV. This is particularly true in age groups >2 years, as these children likely previously received OPV and have waned mucosal immunity (waning estimated at a median rate of 1 year). Thus, in addition to IPV having a direct effect (albeit modest) on intestinal mucosal immunity (through reducing duration, quantity and frequency of excretion), IPV can also boost intestinal mucosal immunity in OPV-vaccinated children. The effect does not appear to be IPV-dose-dependent [48]. The available evidence on IPV mucosal immunity was reviewed [32,33,34].

The second area explored the limits of how few doses of IPV were needed to induce protection against poliomyelitis. This area of work focused on two aspects: (1) number of doses of IPV; and (2) potency (antigen content) of IPV (fractional-dose IPV, fIPV, i.e., one-fifth of a full IPV dose). To understand these approaches, one has to remember that the original formulation work of IPV was conducted in Northern Europe and North America where it had to overcome the interference of maternally derived antibody against the “wild” poliovirus [4,7]. Swartz demonstrated that a higher-potency IPV could overcome the interference of maternally derived antibody [49]. Our work focused on the reduction in number of IPV doses and on its potency (i.e., fIPV), and was building on the studies conducted earlier in India [50,51]. Many follow-up studies were conducted in subjects that had not received OPV, in Cuba [39,40,46], the Philippines [52], the Netherlands [53], Oman [41], Sri Lanka [35], The Gambia [54], Ecuador [55], India [56], Pakistan [42,44] and Bangladesh [24,36,38,57,58] with vaccines from several manufacturers (Staten Serum Institute, Denmark, Bilthoven Biologicals, the Netherlands, and Sanofi Pasteur, France). These studies demonstrated that two fractional IPV doses given at age 14–16 weeks and 8–9 months could induce high seroconversion rates (above 90%). The India fIPV schedule with doses administered at 6 and 14 weeks was evaluated and compared versus a schedule of 10 and 14 weeks, attempting to answer the question of which is more important, longer interval or older age, at first vaccine administration. A 6- and 14-week schedule was significantly more immunogenic [59].

These studies also demonstrated that most infants who did not seroconvert after IPV were immunologically primed against polioviruses [40,57,58,59]. Further studies in Bangladesh and Nepal demonstrated that a single dose of IPV induced immunity against type 2 after 28 months in almost 100% of vaccinees (seroconversion and priming), and that a dose of IPV administered at an age of ≥9 months immunized almost 100% of vaccinees (seroconversion) while inducing a high titer against poliovirus (type 2) [36,37]. The IPV is effective even in countries where OPV efficacy is negatively affected by tropical enteropathy [59]. This condition somehow interferes with intestinal mucosal barrier integrity [60] and is likely responsible for the poor immunogenicity of OPV in some tropical countries [60,61,62,63]. The IPV findings were summarized [32,33,34,38,64] and strengthened the initial recommendation for a single dose of IPV for risk mitigation after the withdrawal of OPV2 from tOPV [5] (Figure 2).

The third area attempts to answer one important unanswered question. Can exclusive IPV use in RI and SIAs induce herd immunity in high force-of-infection tropical countries? In moderate climate countries, IPV appears to induce herd immunity [57]. Indonesia tried to answer this question in a 5-year IPV-only demonstration project in Yogyakarta Province. Environmental surveillance demonstrated that high IPV coverage in RI prevented the circulation of Sabin poliovirus introduced from adjacent OPV-using provinces [28]. After the demonstration project ended, the province continued to use an all-IPV schedule. No evidence of emergence (or detection) of cVDPV2 has been found, despite an outbreak of cVDPV2 affecting Sumatra and Java (12 AFP cases with cVDPV2) in Indonesia (WHO data as of 21 January 2024)

In addition, after 2016, some tropical countries introduced all-IPV schedules in RI, the most important being Malaysia which reported high coverage with a three-dose IPV schedule (>98% nationwide). Sabah Province, Malaysia, a relatively low IPV coverage province and close to Mindanao, Philippines, experienced cVDPV1 and cVDPV2 outbreaks in 2019 and early 2020 with cVDPV1 and cVDPV2 introduced from the Philippines [29]. The outbreaks were controlled rapidly with two rounds of monovalent type 2 OPV (mOPV2) and two rounds of bOPV. No transmission to other provinces on the Island of Borneo or to the Malayan Peninsula were observed.

## 3. Translating New Insights and Evidence into Programmatic Action

As a response to the increasing issue with OPV safety, countries investigated new schedules and vaccines to prevent vaccine-associated paralytic poliomyelitis (VAPP) [10]. As polio eradication made rapid progress, the rare cases of VAPP were becoming increasingly unacceptable to parents. The Western Hemisphere was certified free-of-poliomyelitis in 1994 [65]. In 1997, the United States adopted a sequential schedule of two doses of IPV followed by two doses of OPV, and then an all-IPV schedule in 2000 [66]. Other countries followed a similar path or went directly to all-IPV schedules for routine immunization.

In 2012, the SAGE recommended the inclusion of ≥1 dose of IPV in all OPV-using countries, primarily for risk mitigation in anticipation of OPV cessation [67]. OPV cessation was implemented in April 2016 in a phased manner, with the withdrawal of Sabin type 2 from the trivalent OPV [11,13]. However, IPV supply constraints required the rationing of IPV to the highest risk countries, and only 4–5 years later, all countries received sufficient IPV for routine immunization and catch-up vaccination of the 43 million children who had not received a single dose of IPV.

By 2016, approximately 50 countries, categorized primarily as high-income, relied on IPV exclusively for poliomyelitis prevention, many using combination vaccines with IPV–diphtheria–tetanus–pertussis vaccines (IPV-DTP), some of which included Hemophilus type b and Hepatitis B in a hexavalent formulation [Figure 1].

In 2020, after the IPV supply constraints were resolved, the SAGE recommended a second dose of IPV to close remaining immunity gaps, boost titers, and ensure long-term protection against poliovirus type 2 [15]. The first dose of IPV was recommended with the third dose of pentavalent vaccine (DTP, Hemophilus type b, and Hepatitis B) at age 14 weeks in countries using the Expanded Program on Immunization (EPI) schedule. Some countries, primarily on the India Subcontinent (Bangladesh, India, Nepal, Sri Lanka), as well as Cuba and Ecuador, are using or used a two-dose fractional IPV schedule after OPV2 cessation, with doses administered in India at 6 and 14 weeks and with a third dose added to the visit for measles vaccine at 9 months [68]. Responding to the 2020 SAGE recommendation, India now requires three doses of fIPV administered at ages 6 and 14 weeks and 9 months.

In June 2023, GAVI opened a window for eligible countries to apply for support of hexavalent (pentavalent plus IPV) vaccines, the discussions on an appropriate schedule are under way [68]. The hexavalent vaccine, recommended by WHO, must contain the whole-cell pertussis vaccine. To provide the most immunogenicity for all hexavalent components, a four-dose schedule appears to be necessary with doses given at 6, 10, 14 weeks and 12–23 months.

**Figure 3:**
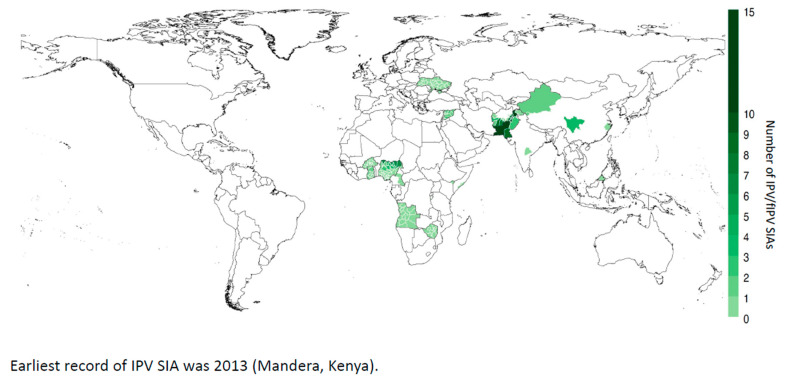
IPV used in campaigns to control cVDPV2 since the switch in 2016.

In addition to its role in RI, IPV (and fIPV) has been used in supplementary immunization activities (SIAs) to eradicate type-1 wild poliovirus (WPV1) in endemic countries of Pakistan, Afghanistan and Nigeria (now non-endemic), as a way to close humoral immunity gaps, especially in densely populated areas (Figure 3). Given the benefit of IPV in effectively preventing paralysis and its impact in boosting mucosal immunity in previously OPV-exposed populations, it was initially included as a tool for cVDPV2 outbreak response following OPV2 withdrawal. Due to global shortages of IPV supply, its use was prioritized for WPV1 endemic countries and, from 2017, no longer supported for cVDPV2 outbreak response. Since OPV2 withdrawal, IPV SIAs have been limited in response to cVDPV2, with use in only 14 countries, 4 of which were due to delayed IPV introduction in RI (e.g., Angola, Ghana, Burkina Faso, and Zimbabwe), and 3 were in WPV1 endemic (Pakistan, Afghanistan) or recently endemic countries (Nigeria). Despite the availability of dose-sparing strategies, fIPV SIAs were only conducted in response to aVDPV2s in Hyderabad, India [69] and Hyderabad, Pakistan [45,70].

Now that supply constraints have been addressed, IPV has again been recommended in cVDPV2 outbreak response, to be used in conjunction with OPV2. Most recently, the SAGE recommended that IPV campaigns be considered in areas with difficulties to achieve and maintain high population immunity (consequential geographies) [71]. Additionally, a recent report suggested that it is feasible to administer fIPV in house-to-house campaigns in one of the most difficult areas to eradicate polio in Northern Nigeria [43]. Previous fIPV campaigns using jet injectors demonstrated great success in terms of feasibility, community acceptance and achieved coverage in Cuba and Pakistan [40,42,45,69]. However, questions remain regarding whether to expand fIPV or IPV in campaigns to control poliomyelitis [72,73].

As new scientific information became available, the technical oversight committee of WHO continued to finetune their recommendation for IPV use, clearly demonstrating that IPV has an increasingly larger role to play in inducing immunity and protecting the world’s children from poliomyelitis. It is also assumed that IPV will assist in eradicating poliovirus from these populations (Table 1).

## 4. Expanding the IPV Supply Infrastructure for Global Demand

To introduce a new vaccine is a massive undertaking which requires readiness investigation, training of health staff, ensuring adequate cold chain capacity and a public information campaign. It also requires the availability of sufficient vaccines for a stable supply chain [74].

In the case of IPV, the GPEI recognized, as early as 2008, that the existing manufacturing base in Europe and North America was insufficient for the requirements of the developing world. While the traditional manufacturers relied exclusively on wild-type poliovirus serotypes to produce IPV, the containment considerations forced the GPEI to evaluate other options (equally immunogenic, but safer to produce). This directly led to a decision to set up a Sabin-IPV Technology Transfer Project [75].

As is well known, the traditional IPV producers handle massive quantities of wild-type polioviruses during production. The potential for accidental leaks or exposures becomes particularly worrisome in a polio-free world. Therefore, the Global Action Plan IV (GAPIV) on poliovirus containment mandates that all facilities handling the poliovirus, including those for vaccine production, adhere to strict bio-containment guidelines [76]. The plan outlines primary (facility containment), secondary (population immunity) and tertiary (facility location) safeguards. These requirements do, however, restrict the number of IPV producers post-eradication, posing significant obstacles to local manufacturers in developing nations.

Therefore, some manufacturers have shifted toward the production of IPV using the Sabin poliovirus strains, which present fewer biosafety risks. A few producers have successfully demonstrated the viability of this approach. Consequently, Sabin-IPV (S-IPV) has been licensed in various presentations in Japan in 2012, China starting in 2012 (multiple producers) and Korea in 2020 [77,78].

In 2008, the WHA resolved to allow for safer IPV production processes, focusing on affordability and the potential development of S-IPV for lower to middle-income countries [79]. Consequently, a partnership was formed between the WHO and Intravacc (formerly part of the Netherlands Vaccine Institute [NVI]) to facilitate the technology transfer of Sabin IPV production technology.

Intravacc functioned as the central technology transfer hub, providing standard operating procedures (SOPs) for manufacturing and generating GMP materials for Phase I/IIa clinical studies, providing production seed strains, and facilitating technology transfers to manufacturers in the developing world. WHO, on the other hand, conducted clinical studies and led the selection of technology transfer recipients, established the expert advisory panel and aided the pre-qualification and licensure process.

Between 2010 and 2012, the WHO disseminated three calls for expressions of interest to vaccine manufacturers in developing countries, aiming to identify two suitable recipients each year for the S-IPV production technology transfer. Following a rigorous selection process that included a multitude of criteria, six entities were chosen by an independent selection committee. The BMGF funded Intravacc’s contribution to the project, while the technology recipients bore the cost of their participation without WHO financial support.

From six producers selected for the S-IPV technology transfer, three achieved national licensure (Sinovac, China; LG Chem, Korea; Beijing Minhai, China) with one (LG Chem) supplying the international market. It required approximately 10 years from first technology transfer selection in 2010 to WHO-prequalification for United Nations purchase. Currently supplied at Euro1.25 per dose in a five-dose formulation to UNICEF, it is the most economical IPV vaccine option [80,81,82].

Presently, there are 10 WHO-prequalified presentations of IPV from five manufacturers (Sanofi, France; Serum Institute of India [SSI], India; AJ Vaccines, Denmark, Bilthoven Biologicals, Netherlands, and Sanofi Healthcare, India), and four prequalified presentations of S-IPV from three manufacturers (Beijing Institute of Biological Products, China; Sinovac, China; LG Chem, Korea) (Table 2). In addition, one hexavalent vaccine by SII, India containing IPV and whole-cell pertussis vaccine is (and another one by Panacea Biotec, India is expected to be shortly) under evaluation for prequalification The hexavalent base will expand massively in the next 3–5 years as countries are interested to introduce this new vaccine that eliminates injections and protects against six antigens.

## 5. Developing the Next-Generation Polio Vaccine

Currently available IPVs (Salk and Sabin) have a successful manufacturing and regulatory track record and are widely used and accepted, as they are very safe to use in the field, but they have two drawbacks: (1) they require large quantities of live virus in the manufacturing facilities before they are chemically inactivated, which poses a threat should containment of these facilities be breached and, (2) they do not directly induce mucosal immunity and thus, while they efficiently protect those who receive the vaccine, they do not prevent the vaccine recipients from shedding the virus initially, but reduce the proportion of vaccinees excreting, the length of excretion and the titer of virus excreted (Table 1).

The GPEI is thus interested in developing vaccines that are not affected by the drawbacks mentioned, with the highest importance being set on responding to growing containment requirements.

Two major development projects are currently being undertaken:

1. Poliovirus Virus-Like particles (PV VLPs)

This project was launched by WHO in 2010 with the aim of developing PV VLPs as potentially ideal vaccines for the post-eradication era to eventually replace IPVs. Vaccines based on VLPs are already established and successful for other diseases, such as cervical cancer caused by human papillomavirus [83]. WHO contracted a research and development consortium under the lead of the University of Leeds, UK, with this task.

By definition, VLPs are particles that closely resemble viruses, but are non-infectious as they do not contain any viral genetic material. PV VLPs thus do not contain poliovirus RNA. This exempts them from containment requirements, which will only become stricter as the program achieves polio eradication.

Native PV capsid proteins do not form a stable particle in the absence of RNA and thus PV VLPs require genetically modified capsid proteins plus addition of stabilizing elements such as pocket factors [84].

The consortium was able to generate thermostable VLPs that are D-antigenic and have a structure that is indistinguishable from Polio WT virus from a number of expression systems such as plants [85], mammalian cells [86], yeast [87,88,89,90,91] and baculovirus. Preclinical immunogenicity studies of PV VLPs adjuvanted with alum [Al(OH)_3_] in Wistar rats, the “gold standard” for IPV lot release testing, show similar or better immunogenicity compared to IPV, which is encouraging.

In 2019, WHO published a call for Expression of Interest to find partners for the commercialization of PV VLPs. This call was answered by a large number of interested parties, mainly commercial manufacturers. Following advice of an independent advisory panel, WHO chose some promising applicants with whom collaboration is currently ongoing. The focus is set on yeast and baculovirus as the two expression systems that will most likely allow production of goods at low cost. In addition, the Bill and Melinda Gates Foundation recently issued a request for proposal to selected potential partners for which the final selection is imminent.

The target product profile for PV VLPs, very similar to the one for IPV, calls for PV VLPs as a standalone presentation as well as a component for multivalent vaccines such as hexavalent vaccines. The main use, with an affordable market price as a necessary requirement, is anticipated for RI with a potential for outbreak response as well. The VLPs development is continuing and is now in Phase 1 of clinical development.

In addition, in the quest to develop a poliovirus vaccine presentation that could potentially avoid the need for a cold chain, which would not require highly trained vaccinators as with IM injections and would potentially be dose-sparing (thus cost-effective) and even providing mucosal immunity, the consortium collaborates with the University of Queensland, Australia, to develop microneedle patches (MNPs) with or without the addition of including double-mutant heat-labile enterotoxin of *Escherichia coli* (dmLT) [17,18,92], an adjuvant that has been shown to induce mucosal immunity in other applications.

2. S19 IPV

Following WHO’s recommendation for the use of attenuated Sabin strains for IPV production, to avoid or reduce the mentioned risks that Salk and Sabin IPV pose regarding containment, the former National Institute for Biological Standards and Control, UK (NIBSC) now integrated into the Medicines and Healthcare products Regulatory Agency, UK (MHRA) developed a strain (S19) which is hyper-attenuated, genetically stabilized and most probably cannot replicate in people (i.e., S19 cannot replicate at human body temperature) and are also highly restricted in their potential to revert to a neurovirulent form during the manufacturing process. The strain has been shown to be as immunogenic as the current IPV and can be produced on a large scale [93].

S19 strains have obtained a (time-limited) waiver from containment requirements for manufacturing by the Containment Advisory Group (CAG) [94]. This S19 strain is already used for neutralization assays and is replacing the infectious Sabin strains, and S19-based IPV is in development by one manufacturer for a hexavalent presentation which is currently in Phase 1 clinical development and potentially for a standalone adjuvanted presentation.

The WHO SAGE in October 2022 stated “SAGE recognized the importance of clinical research and product development and encouraged accelerated efforts to develop, license and commercialize …, VLP and other non-infectiously manufactured IPV-like vaccines, …”.

The two projects outlined in this section aim to fulfill this guidance. Poliovirus VLPs have just entered Phase 1 of clinical development, but the development is by nature unpredictable as to success and associated timelines, and an optimistic timeline for successful commercialization by manufacturers obtaining national licensure and WHO prequalification could be envisaged by 2028.

VLPs have a theoretical advantage over S19 strains, as by definition they cannot replicate in humans and thus cannot revert to a neurovirulent form, whereas S19 would still need to be proven by studies in humans to have the same properties.

## 6. Conclusions and Future Perspectives

Much has been learned over the past two decades regarding the efficacy, strategic use for programmatic action and safe production of IPV. Since the 2016 switch from tOPV to bOPV, and the concomitant introduction of ≥1 dose of IPV into RI schedules, IPV became a truly global vaccine, benefitting all children regardless of residing in industrialized or developing countries.

IPV has demonstrated versatility. In industrialized countries, most countries use combination pentavalent or hexavalent formulations with IPV [4,95]. In developing countries, countries use stand-alone IPV or fractional-dose IPV. This will change in the next three to five years after GAVI opens a window for support of combination hexavalent vaccines with IPV that use whole-cell pertussis vaccine [68].

For outbreak control, stand-alone IPV and/or fIPV will also be increasingly considered for campaigns use [71]. An evaluation in Northern Nigeria demonstrated the feasibility of fIPV house-to-house campaigns using a needle-free injection device [43]. A recently published case–control study in Nigeria reported a single-dose IPV vaccine effectiveness of almost 90% [47]. This report, the feasibility of house-to-house administration of fIPV [43,45,69], the superior acceptance of an injectable vaccine, particularly fIPV [39,45,56,70] administered with a needle-free device and the recent recommendation of IPV campaign use in consequential geographies [71], suggests that a new strategy to reach and surpass the threshold for herd immunity, especially targeted to difficult areas that may include Northern Nigeria, Eastern Democratic Republic of the Congo (DRC), Somalia and Yemen. In these areas, annual supplemental IPV house-to-house outreach to children <5 years with high coverage (in addition to routine polio vaccine use) could both interrupt cVDPV2 transmission, prevent the emerge of new cVDPVs and most importantly massively reduce the paralytic disease burden. Further innovative thinking is needed to optimize such extended outreach use, especially weighing the cost versus population immunity gain, and increasing quality and coverage (Table 2).

The Sabin-IPV technology transfer project has been successful. At least two manufacturers obtained national licensure, and one manufacturer has obtained WHO-prequalification for supply to United Nations agencies. The entry of LG Chem has disrupted the IPV pricing structure and resulted in massive savings for the global support base (GAVI-supported countries) since 2021 [82].

However, the further development of IPV must eventually result in non-infectious production methods, the cracking of the “holy grail” of non-infectious polio vaccines inducing mucosal immunity, and better methods for administering injectable vaccines in house-to-house campaigns. The development of virus-like particles (VLPs) is encouraging, the S19-based Sabin virus is already used as challenge virus for safer neutralization assays and S19-based IPV may add yet another level of safety for meeting production containment requirements [92,93].

The world has benefitted from two excellent vaccines for polio prevention, IPV and OPV. But neither of these vaccines are perfectly suited for achieving eradication. Whether IPV-induced intestinal mucosal immunity can achieve herd immunity in tropical countries with high force of poliovirus transmission has not been answered conclusively, but findings from Indonesia and Malaysia are encouraging [28,29]. To harvest the full potential of IPV, high vaccination coverage is necessary [95]. OPV use, however, induces mucosal immunity but constantly introduces new live viruses into communities that can mutate and/or recombine to source new endemic and epidemic transmission (i.e., “fighting fire with fire”). This seeding cycle needs to be broken.

Looking into the future, it seems obvious that all infectious poliovirus vaccines must be discontinued to achieve eradication, and that the world must rely solely on non-infectious polio vaccines for maintaining high population immunity. To get to this point, a massive program-of-work will be required, but the rewards will also be massive. Polio eradication will finally be within our grasp.

## Figures and Tables

**Table 1:** IPV vaccine effect on humoral and mucosal immunity.

IPV Effect	Advantages	Geographic Focus	References
Induces humoral immunity	Provides life-long immunity from paralytic poliomyelitis	Globally	Francis [3], Vidor [4]
Closes humoral immunity gaps in previously IPV- or OPV-vaccinated individuals	Prevents paralytic disease after poliovirus exposure	Globally	Vidor [4], Dang [25]
Boosts humoral antibody levels	Can reduce excretion of poliovirus after infection	Globally	Marine [26], Sutter [7]
Generates herd immunity	Less than 100% IPV coverage seem to induce herd immunity	Shown in countries with good hygiene and sanitation–size of effect unknown in high force-of-infection communities, but possibly in Indonesia and Malaysia	Stickle [27], Wahjuhono [28], Snider [29]
Induces pharyngeal mucosal immunity	Reduces oral-to-oral transmission of poliovirus	Mode of transmission more important in industrialized countries	Sutter [7], Onorato [19], Fine [21]
Induces a “modest” degree of mucosal intestinal immunity	Shorter excretion, decrease viral output in stool, and lower frequency → reduction of 95% in excreted poliovirus virions over course of infection	More important in countries with good hygiene and sanitation	Sutter [7], Ghendon [20], Fine [21], Brickley [22], Cuba IPV Collaborative Study Group [23]
Boosts intestinal mucosal immunity in OPV-vaccinated individuals	Reduces fecal-to-oral transmission of poliovirus–from 39 to 76% depending on age	More important in tropical developing countries	John [30], Jafari [31], Snider [24,29], Hird [32], Parker [33], Macklin [34], Gamage [35]
Later administration (age ≥3 mos) overcomes interference of maternally derived antibody	A single dose at 9 mos can lead to almost 100% seroconversion	Globally	Aziz [36], Sharma [37], Anand [38]
Induces priming immune responses in non-responders	A second dose can effectively boost humoral immunity rapidly <7 days	Globally	Resik [39,40], Mohammad [41]
Priming immune response duration	Demonstrated to persist at least 28 mos	Globally	Sharma [37]
Immunogenicity not affected by tropical enteropathy	IPV immunogenic in populations affected by tropical enteropathy (in contrast to OPV)	More important in tropical developing countries	Saleem [42]
IPV can be administered in house-to-house campaigns	Demonstrated in SIA in Northern Nigeria → may substantially increase coverage	More important in areas with suboptimal health infrastructure	Biya [43]
Fractional-dose IPV (fIPV) can reduce cost of polio immunization schedule or use in campaigns	Two doses of fIPV can induce nearly 100% immunity if given in appropriate schedule but only uses 40% of a full dose of IPV	More important for countries not currently supported by GAVI, such as India	Aziz [36], Sharma [37]
Administration of fIPV with intradermal jet injector Tropis®	Facilitates intradermal administration and results in dose saving	More important in house-to-house campaigns attempting to reach a high proportion of the target population	Bullo [44], Pervaiz [45], Resik [46]
Single-dose IPV vaccine effectiveness close to 90% in Nigeria	Should lead to re-evaluation of strategy to achieve and maintain population immunity in “consequential geographies”	More relevant to tropical areas with suboptimal health infrastructure	Cooper [47]

**Table 2:** IPV producers and products with World Health Organization (WHO)-prequalification for United Nations purchase.

Seed Strains	Manufacturers	WHO Pre-Qualification Date	Presentation
Wild poliovirus	Sanofi S.A., Paris, France	9DEC2005 19DEC2014	10-dose IPV DTaP-HBV-Hib-IPV
	Bilthoven Biologicals B.V., Bilthoven, The Netherlands (a subsidiary of SII)	6DEC2010 28NOV2014	1-dose IPV 5-dose IPV
	AJ Vaccines A/S, København, Denmark	23DEC2010 21APR2020	1-dose IPV 5-dose IPV
	Serum Institute of India (SII), Pune, India (IPV from Bilthoven Biologicals B.V.)	28OCT2016 11JUL2019	1 + 2 + 5-dose IPV 10-dose IPV
	Sanofi Healthcare India Private Ltd., India (with IPV from Sanofi S.A., France)	01OCT2018 22APR2022	5-dose IPV 10-dose IPV
Sabin strains	Sinovac Biotec Co., Ltd., Beijing, China §	06JUN2022	Single dose IPV
	LG Chem Ltd., Seoul, Republic of Korea §	01JUN2021 21DEC2020	Single dose IPV 5-dose IPV
	Beijing Institute of Biological Product Co., Ltd., Beijing, China	15FEB2022	Single dose IPV

§ Participants of INTRAVACC/WHO Sabin-IPV technology transfer project.
